# Reconsidering the Perinatal Risks of Maternal Underweight in Japan

**DOI:** 10.2188/jea.JE20260021

**Published:** 2026-08-05

**Authors:** Jiayi Chen

**Affiliations:** Department of Stomatology, Suzhou Wujiang District Hospital of Traditional Chinese Medicine, Suzhou, China

Dear Editor,

I read with great interest the recent article by Kobayashi et al, entitled “Impact of Maternal Underweight on Infant and Maternal Outcomes in Japanese Women: A Systematic Review and Meta-analysis.”^1^ The authors should be commended for conducting a comprehensive and timely synthesis of evidence addressing an often-overlooked public health issue in Japan.

This review is particularly valuable given the uniquely high prevalence of pre-pregnancy underweight among Japanese women of reproductive age. By systematically analyzing 34 studies, the authors provide robust evidence that maternal underweight is consistently associated with adverse perinatal outcomes, including low birth weight, small for gestational age, and preterm birth. The finding that these risks persist despite the high background prevalence of underweight in Japan is especially noteworthy and challenges the assumption that population norms may attenuate biological risk.

The methodological rigor of this study is a key strength. Adherence to PRISMA 2020 guidelines, registration in PROSPERO, and the inclusion of both international and Japanese databases enhance the transparency and completeness of the review. In addition, the authors’ sensitivity analyses across different body mass index definitions strengthen confidence in the robustness of their conclusions.

At the same time, the study highlights important areas for future research. Residual confounding remains a concern, as many included studies lacked adjustment for key variables, such as maternal age, socioeconomic status, and smoking. Further prospective studies incorporating standardized exposure measurements and detailed covariate adjustment would help clarify causal pathways. Moreover, stratified analyses by age group or severity of underweight may provide additional insights relevant to targeted interventions.

Overall, this meta-analysis makes a significant contribution to the literature and has important implications for preconception counseling and public health policy in Japan. Increasing awareness of the risks associated with pre-pregnancy underweight should be considered an essential component of maternal and child health strategies.
